# Novel bimetallic Cu/Ni core-shell NPs and nitrogen doped GQDs composites applied in glucose in vitro detection

**DOI:** 10.1371/journal.pone.0220005

**Published:** 2019-07-22

**Authors:** Shuyao Zhang, Zheling Zhang, Xiaoling Zhang, Jian Zhang

**Affiliations:** 1 School of Materials Science and Engineering and Guangxi Key Lab for Informational Materials, Guilin University of Electronic Technology, Guilin, Guangxi, P. R. China; 2 Key Laboratory of Cluster Science of Ministry of Education, Beijing Key Laboratory of Photoelectronic/Electrophotonic Conversion Materials, Analytical and Testing Center, School of Chemistry and Chemical Engineering, Beijing Institute of Technology, Beijing, P. R. China; Kwangwoon University, REPUBLIC OF KOREA

## Abstract

In present work, a highly sensitive biosensor with high selectivity for glucose monitoring is developed based on novel nano-composites of nitrogen doped graphene quantum dots (N-GQDs) and a novel bimetallic Cu/Ni core-shell nanoparticles (CSNPs) (Cu@Ni CSNPs/N-GQDs NCs). With the tuned electronic properties, N-GQDs helped bimetallic core-shell structure nanomaterials from aggregation, and separate the charges generated at the interface. This novel nano-composites also have the good electrical conductivity of N-GQDs, catalyst property of Cu/Ni bimetallic nano composite, Cu@Ni core-shell structure and the synergistic effect of the interaction between bimetallic nano composite and N-GQDs. While modified the electrode with this novel nano-composites, the sensor’ linear range is 0.09 ~ 1 mM, and the limit of detection (LOD) is 1.5 μM (S/N = 3) with a high sensitivity of 660 μA mM^-1^ cm^-2^, and rapid response time (3 s). Its’ LOD is more than 74 times lower than the traditional Cu@Ni CSNPs modified working electrode. It also has higher sensitivity and wider linear range. This indicates the great potential of applying this kind of nano composites in electrode modification.

## Introduction

With the rapid development of nanotechnology, a lot of carbon materials have emerged in recent years, such as fullerenes, multi-wall carbon nanotubes, carbon nanofibers, graphene[1], graphene quantum dots (GQDs) and so on. These carbon materials show unique physical and chemical properties. Among these materials, GQDs[2, 3] are graphene sheets with only monolayer or less layers. They are a kind of zero dimensional carbon materials with sizes less than 10nm. They usually contain functional groups, e.g. carboxyl, hydroxyl, carbonyl, epoxide etc. at the edges[4]. They were popular reported for their high biocompatibility, small sizes and low costs. Quantum confinement and edge effects endow them with optical activity, conductivity and chemical inertness. GQDs exhibit strong photoluminescence (PL), excellent electrochemiluminescence (ECL) and electrochemical activity because they are favorable electron donors and acceptors due to their large surface area and abundant edge sites. Surface chemistry also applied to GQDs synthesis and makes the interesting properties available.

Owing to these properties, GQDs have gained wide attention for their enormous potential in varies applications. For instance they are widely applied in photovoltaics, organic light emitting diodes, fuel cells, photocatalysis, bioimaging, biosensing, biomedicine, environmental monitoring, thermal interface materials etc[5–8]. Biosensor is one of the most interesting application[8–12]. Combining with abundant detection methods, scientists designed varies analytical strategies based on GQDs’ unique properties. Among all the biosensors, glucose sensors, especially glucose electrochemical sensors, are widely studied for very long history[13–16]. Because glucose is a very important carbohydrate and play irreplaceable role for organisms. It is also the index for diabetes which is one of the most common deadliest diseases and it is affecting by several millions of people all over the world.

On the other hand, various nano-structured metals, alloys and metal oxides have been extensively applied in non-enzymatic electrode modification, due to their increased specific surface area, rapid mass transport, significant catalytic activity and so on[17–22]. Among those nanomaterials, nickel and copper based nanomaterials have significant electrocatalytic effect on glucose[23–36]. For instance, bilayer Ni/Cu porous nanostructured film[37], Ni-Cu/TiO_2_ NTs[38], Ni/Cu/MWCNT electrodes[39] are all prepared by different methods, and show higher sensitivity for the quantitative determination of glucose than single-metal composite. This type of composites have large exposed area and excellent diffusion properties[37–39]. On this point, the core-shell nanostructure of Ni/Cu bimetallic have attracted researchers’ attention for glucose sensing[1].

In this work, the novel bimetallic Cu/Ni and N-GQDs nano-composites (Cu@Ni CSNPs/N-GQDs NCs) have been synthesized by hydrothermal method and a one-pot solvothermal method. With our gentle synthesis method, the size of N-GQDs can be uniform with the nitrogen content about 12.93%. The size of Cu @ni CSNPs/N-GQDs NCs is 30–80 nm, and N-GQDs is uniformly coated on its surface, with bimetallic Cu/Ni co-catalytic performance and good electrical conductivity. A series of non-enzymatic glucose sensors are constructed with Cu@Ni CSNPs/N-GQDs NCs modified glassy carbon electrode (Cu@Ni CSNPs/N-GQDs/GCE), Cu@Ni CSNPs/GCE and Cu@Ni CSNPs/N-GQDs/GCE. Their electrochemical properties and electrocatalytic activities are compared. It is excited that Cu@Ni CSNPs/N-GQDs/GCE shows the best electrocatalytic performance for glucose oxidation, and displays the lowest detection limit(LOD) 1.5 μM (S/N = 3), a wider linear range from 0.09 mM to 1 mM and high sensitivity 660 μA mM^-1^ cm^-2^. Comparing with the sensor based on Cu@Ni CSNPs modified working electrode, the electrode modified with Cu@Ni CSNPs/ N-GQDs NCs makes the sensor’s LOD more than 74 times lower, higher sensitivity and wider linear range. These indicate that well designed N-GQDs’ composites have great application prospects in improving electron migration rate and electrocatalytic performance of electrode surface.

## Materials and methods

### Chemicals, reagents and characterization

Urea (≥99.0%), N, N-dimethyl formamide (DMF, 99.9%), Copper (II) chloride dihydrate (CuCl_2_·2H_2_O, 99.0%), Nickel (II) chloride hexahydrate (NiCl_2_·6H_2_O, ≥98.0%), NaOH (96.0%), AA (≥99.7%), sucrose, glucose and NaCl (≥99.5%) were purchased from XiLong Scientific. Citric acid (99.5%) and DA (98%) were purchased from Aladdin. 5wt% of Nafion was purchased from Sigma Aldrich. Ethylene glycol (EG, 99.0%), maltose, fructose, D-galactose and UA (99%) were purchased from Sinopharm Chemical Reagent Co., Ltd. Ultrapure water (ELGA, Veolia, 18.2 MΩ) was used throughout the experiments, and all reagents which were not mentioned above were of analytical grade.

The samples were characterized by X-ray photoelectron spectra (XPS, Thermo ESCALAB 250XI), transmission electron microscopy (TEM) and high resolution transmission electron microscope (HRTEM, FEI Tecnai G^2^ 20), ultraviolet-visible spectra (UV-Vis, PerkinElmer Lambda 365), Fourier transform infrared spectra (FTIR, Bruker) and X-ray diffraction measurement (XRD, Bruker D8 Advance). All electrochemical experiments were performed on the electrochemical workstation (Shanghai, CHI 660C). The working electrodes were modified glassy carbon electrode (GCE, 3 mm in diameter), the reference electrode was saturated calomel electrode (SCE), and a platinum wire electrode was usedas the auxiliary electrode.

### Preparation of N-GQDs

N-GQDs were synthesized by hydrothermal method. 1g citric acid and 0.947 g urea weretaken into the Teflon-lined, 10 ml ultrapure water was added and stirred until citric acid and urea fully dissolved. Then put the stainless-steel autoclave into the muffle furnace and heated at 180°C for 5 hours. After the sample was cooled to room temperature, the mixture was filtered and dialyzed against a 3000 D dialysis bag to neutrality, then the solution was dried by a freeze dryer to obtain the powder.

### Preparation of Cu@Ni CSNPs/N-GQDs NCs

The solution contained 0.0335 g N-GQDs and 33.3 ml EG was dissolved and ultrasonically dispersed 5 hours. The solution contained 0.1482 g CuCl_2_·2H_2_O and 0.2 g NiCl_2_·6H_2_O and 33.3 ml EG and the solution contained 0.675 g NaOH and 8.3 ml EG were both prepared by ultrasonically dispersion for 1 hour. Then the solution of CuCl_2_·2H_2_O and NiCl_2_·6H_2_O was added dropwise into the N-GQDs solution under stirring, following with the NaOH solution added dropwise and stirred at room temperature for 1 hour. When the reaction was thoroughly finished, the mixture was transferred to a Teflon-lined and heated at 200°C in the muffle furnace for 5 hours. After the sample was cooled to room temperature, it was stirred for 10 minutes and centrifugalized at 8000 rpm for 5 minutes, then washed repeatedly with ethanol and ultrapure water 3 times and vacuum dried overnight to obtain the black composite powder.

### Preparation of Cu@Ni CSNPs, Cu NPs and Ni NPs

For convenience of comparison, Cu@Ni CSNPs, Cu NPs and Ni NPs were prepared respectively with the same procedure. 0.2557 g CuCl_2_·2H_2_O and 0.3565 g NiCl_2_·6H_2_O and 60 ml EG were dissolved and ultrasonically dispersed for 1 hour. Then the NaOH solution mentioned was added dropwise into the above solution, and stirred at room temperature for 1 hour. While the reaction thoroughly finished, the mixture was heated at 200°C in the muffle furnace for 5 hours. After the mixture was cooled at room temperature, it was stirred for 10 minutes and centrifugalized at 8000 rpm for 5 minutes, then washed repeatedly with ethanol and ultrapure water respectively. Finally, the mixture was vacuum dried overnight to obtain the black Cu@Ni CSNPs powder. By the same conditions, the Cu NPs and Ni NPs were also prepared.

### Preparation of the modified GCE

40 g DMF and 4 g Nafion solvent were taken and ultrasonically dispersed for 1 hour. 0.02 g Cu@Ni CSNPs/N-GQDs NCs was dispersed into 20 ml DMF and Nafion mixture, then ultrasonically dispersed for 1 hour to obtain catalyst suspension. After the GCE preparation, 10 μL catalyst suspension was coated on the working electrode. Then the electrode dried under infrared lamp to obtain the modified Cu@Ni CSNPs/N-GQDs/Nafion/GCE. For comparison, the Cu@Ni CSNPs/Nafion/GCE was also prepared by the same method.

## Results and discussion

### Materials characterization

**Fig 1:**
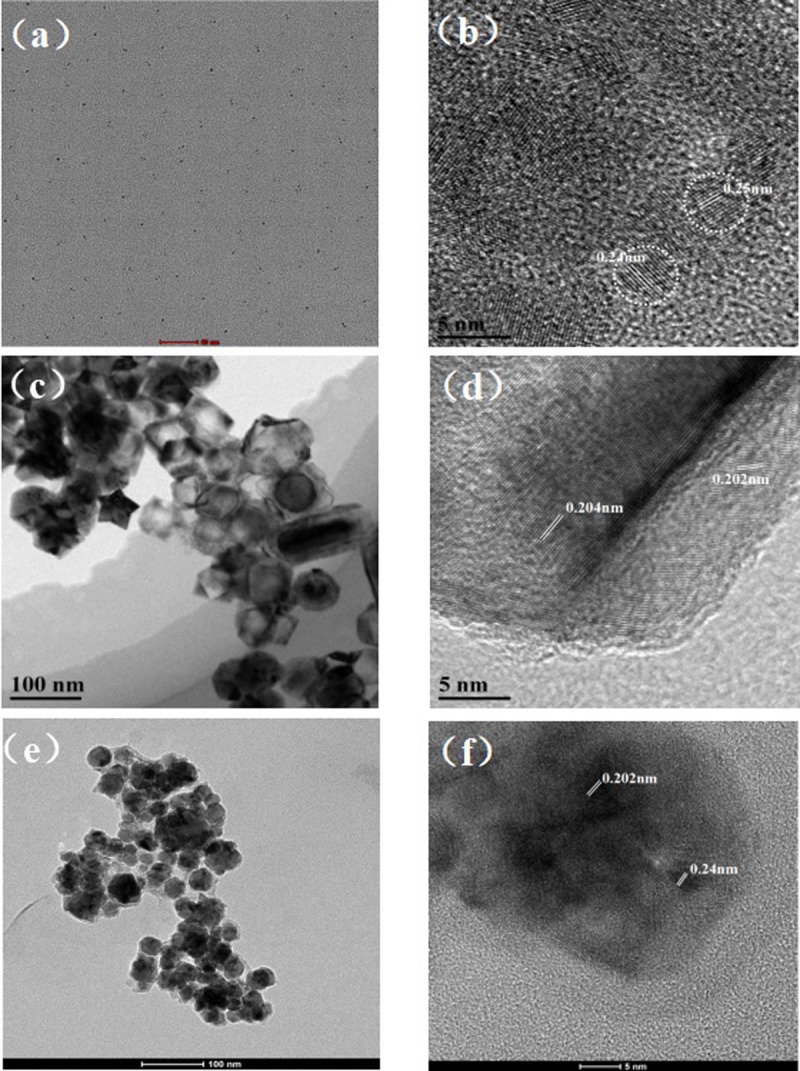
TEM and HRTEM images of N-GQDs, Cu@Ni CSNPs and Cu@Ni CSNPs/N-GQDs NCs. TEM image (**a**) and HRTEM image (**b**) of N-GQDs; TEM image (**c**) and HRTEM image (**d**) of Cu@Ni CSNPs; TEM image (**e**) and HRTEM image (**f**) of Cu@Ni CSNPs/N-GQDs NCs.

The morphology of N-GQDs, Cu@Ni CSNPs and Cu@Ni CSNPs/N-GQDs NCs composites were characterized by TEM and HRTEM. As can be seen from Fig 1A, N-GQDs are “point” shaped and evenly distributed. In Fig 1B, HRTEM image shows that the N-GQDs have average diameter about 5 nm, and the lattice spacingis about 0.24 nm, corresponding to the (100) lattice spacing of the GQDs [40]. Fig 1C is the TEM image of Cu@Ni CSNPs. Because of their double alloy structure, high crystallinity can be clearly seen from the figure. They have size about 50–110 nm, and the average lattice spacing 0.203 nm (Fig 1D), corresponding to the {111} crystal plane of Cu and Ni[1]. Fig 1E is the TEM image of Cu@Ni CSNPs/N-GQDs NCs.The nano composites’ sizes are about 30–80 nm and spherical. From Fig 1F, the lattice spacings are 0.202 nm and 0.24 nm, corresponding to the lattice spacings of N-GQDs, Cu and Ni. S3A–S3C Fig are the mapping maps of Cu@Ni CSNPs/N-GQDs NCs composites, demonstrating the presence of Cu and Ni in the composites. And as can be seen from the figure that the GQDs are coated on the surface of the double alloy.

**Fig 2:**
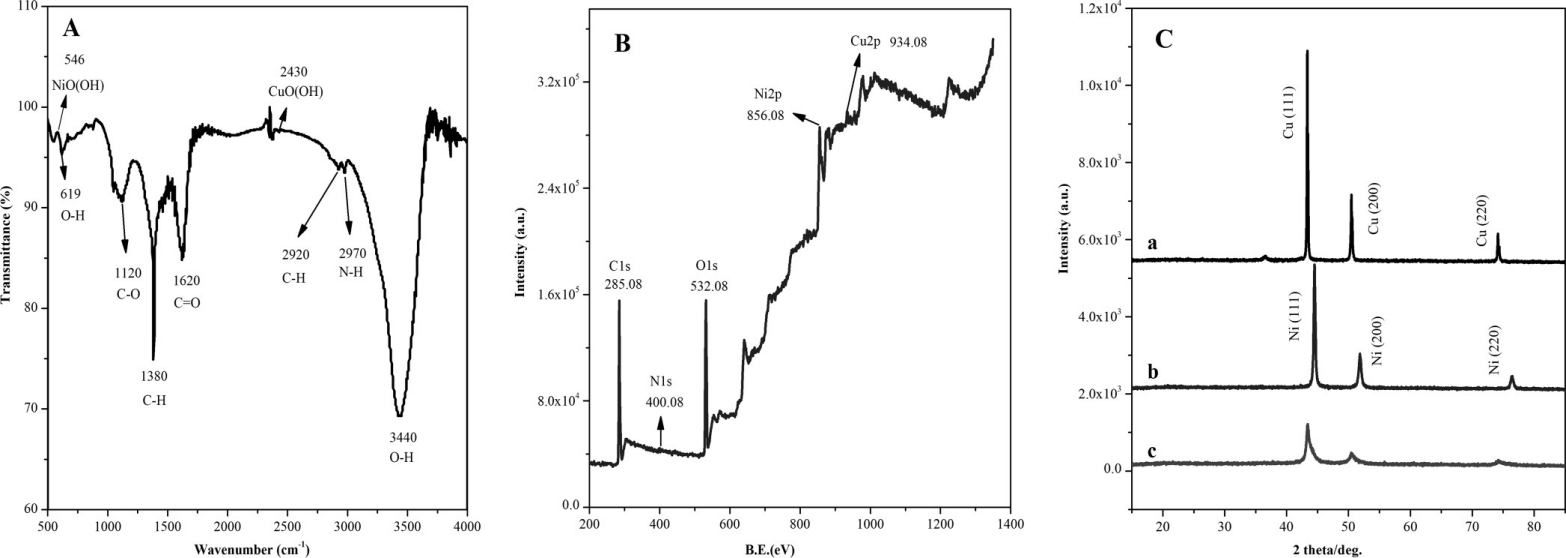
FTIR, XPS and XRD spectra of Cu@Ni CSNPs/N-GQDs NCs. (**A**) FTIR spectrum, (**B**) XPS full spectrum, (**C**) XRD patterns of Cu NPs (**a**), Ni NPs (**b**), Cu@Ni CSNPs/N-GQDs NCs (**c**).

Fig 2A is FTIR spectrum of Cu@Ni CSNPs/N-GQDs NCs. Peaks at ~619 nm and ~3440 nm are stretching vibration peaks corresponding to O-H functional groups, peaks at ~1120 nm, ~1620 nm and ~2970 nm are stretching vibration peaks corresponding to C-O, C = O and N-H functional groups, peaks at ~1380 nm and ~2910 nm are stretching vibration peaks corresponding to C-H functional groups, peaks at ~546 nm and ~2430 nm are characteristic peaks corresponding to NiO(OH) and CuO(OH), respectively. They indicate the presence of Cu and Ni in the composites, and exist in the form of oxides. S1A Fig is the full XPS spectrum of the N-GQDs. ~285.08 eV corresponds to C1s, ~400.08 eV corresponds to N1s, and ~532.08 eV corresponds to O1s. The atomic ratio of C1s: O1s: N1s is about 55.84: 31.22: 12.93 (shown in S1 Table). S1B Fig is the C1s spectrum of N-GQDs, and the peak at ~285.3 eV corresponds to the characteristic peak of C-N/C = N functional groups. S1C Fig is the N1s spectrum of N-GQDs, corresponding to the peaks of pyridinic-N, pyrrolic-N and graphitic-N functional groups at ~399.6 eV, ~400.4 eV and ~401.5 eV, respectively. These indicate that the nitrogen atoms have been successfully doped into GQDs which giving the interior and edge of GQDs more cavitation electron pairs which means the composite is more conducive for electron transmission. S1D Fig is the O1s spectrum of N-GQDs. ~531.3 eV and ~532.5 eV correspond to characteristic peaks of C = O and C-O functional groups, respectively. The presence of oxygen-containing groups makes N-GQDs more soluble in water and organic solvents, and easily to be combined with other materials. It also makes the composite easier to form membrane and broaden the application of quantum dots. XPS is an effective technique for analyzing elemental composition and functional groups type in samples. Fig 2B is XPS full spectrum of Cu@Ni CSNPs/N-GQDs NCs, ~285.08 eV corresponds to C1s, ~400.08 eV corresponds to N1s, ~532.08 eV corresponds to O1s, ~856.08 eV corresponds to Ni2p, and ~934.08 eV corresponds to Cu2p. The atomic ratio of C1s: O1s: N1s: Ni2p: Cu2p is about 63.35: 27.46: 1.11: 7.19: 0.89 (shown in S1 Table). S2A Fig is the C1s spectrum of Cu@Ni CSNPs/N-GQDs NCs, the characteristic peak of C-N functional groups at ~284.88 eV corresponds to the C-N peak of N-GQDs in S1B Fig. S2B Fig is the spectrum of Cu2p, and ~933.18 eV is the characteristic peak of Cu^0^. S2C Fig is the spectrum of Ni2p, and ~855.78 eV is the characteristic peak of Ni^0^. They indicate the presence of Cu and Ni in Cu@Ni CSNPs/N-GQDs NCs composite. Fig 2C is XRD patterns of Cu NPs (a), Ni NPs (b) and Cu@Ni CSNPs/N-GQDs NCs (c). The figure shows the Cu NPs have three sharp characteristic peaks of Cu (111), Cu (200) and Cu (220), the Ni NPs have three sharp characteristic peaks of Ni (111), Ni (200) and Ni (220). The characteristic peaks of Cu@Ni CSNPs/N-GQDs NCs have lower relative peak intensity and wider peak half-width, which is obviously composed of two characteristic peaks of Cu and Ni. It indicates that Cu@Ni CSNPs/N-GQDs NCs composite containing Cu and Ni, and the two parts are present separately.

### Electrochemical behavior of Cu@Ni CSNPs/Nafion/GCE and Cu@Ni CSNPs/N-GQDs/Nafion/GCE

**Fig 3:**
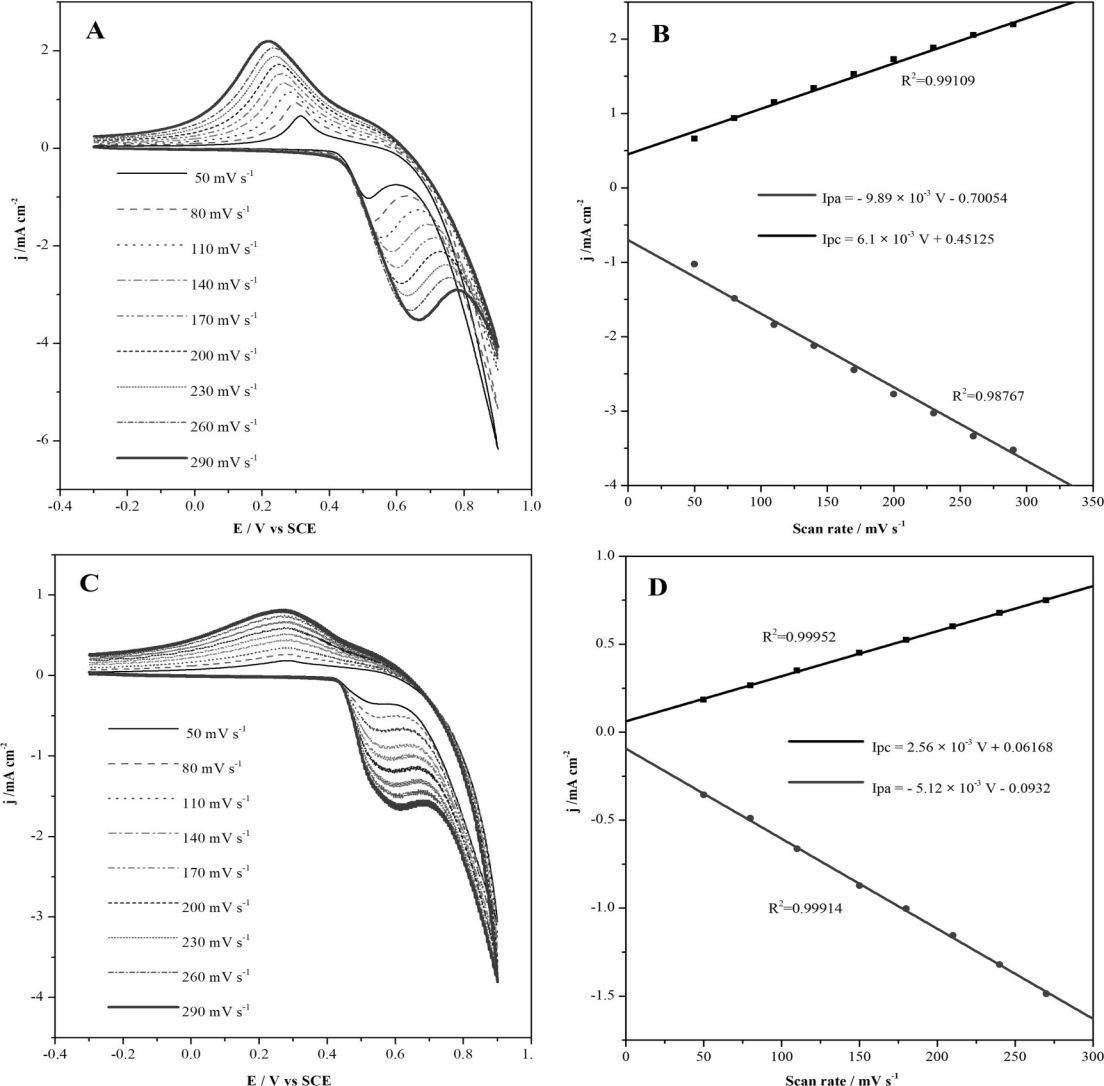
Electrochemical behavior of Cu@Ni CSNPs/Nafion/GCE and Cu@Ni CSNPs/N-GQDs/Nafion/GCE. CVs of Cu@Ni CSNPs/Nafion/GCE in 0.1 M NaOH at different scan rates (**A**) and plot of peak current versus the potential scan rates (**B**). CVs of Cu@Ni CSNPs/N-GQDs/Nafion/GCE in 0.1 M NaOH at different scan rates (**C**) and plot of peak current versus the potential scan rates (**D**).

The modified Cu@Ni CSNPs/Nafion/GCE and Cu@Ni CSNPs/N-GQDs/Nafion/GCE electrodes were placed in cell containing 0.1 M NaOH solution to be tested at different scan rates. As shown in Fig 3A, when the scan rate is increased from 50 mV s^-1^ to 290 mV s^-1^, both anodic and cathodic currents with modified Cu@Ni CSNPs/Nafion/GCE are increased with the increasing of scan rates. The linear relationships are *I*pa = - 9.89 ×10^−3^ v—0.70054 (R^2^ = 0.98767), *I*pc = 6.1 ×10^−3^ v + 0.45125 (R^2^ = 0.99109), as shown in Fig 3B. As shown in Fig 3C, both anodic and cathodic currents of the sensor with modified Cu@Ni CSNPs/N-GQDs/Nafion/GCE are increased with the increasing of scan rates. The linear relationships are *I*pa = -5.12 ×10^−3^ v—0.0932 (R^2^ = 0.99914), *I*pc = 2.56 ×10^−3^ v + 0.06168 (R^2^ = 0.99952), as shown in Fig 3D. Both of them indicate that the electrochemical kinetics of these two electrodes are surface controlled. Compared with these two electrodes, modified Cu@Ni CSNPs/N-GQDs/Nafion/GCE electrode has better linearity and better electrochemical performance. As the scan rate increasing, the anodic peak potential is positive and the cathodic potential is negative, that caused a larger peak-to-peak separation. These results are due to nucleation of NiO(OH) and increased activity centers of Ni^3+^ and Ni^2+^ species[41,42].

### Electrocatalytic oxidation of Glucose on Cu@Ni CSNPs/Nafion/GCE and Cu@Ni CSNPs/N-GQDs/Nafion/GCE

**Fig 4:**
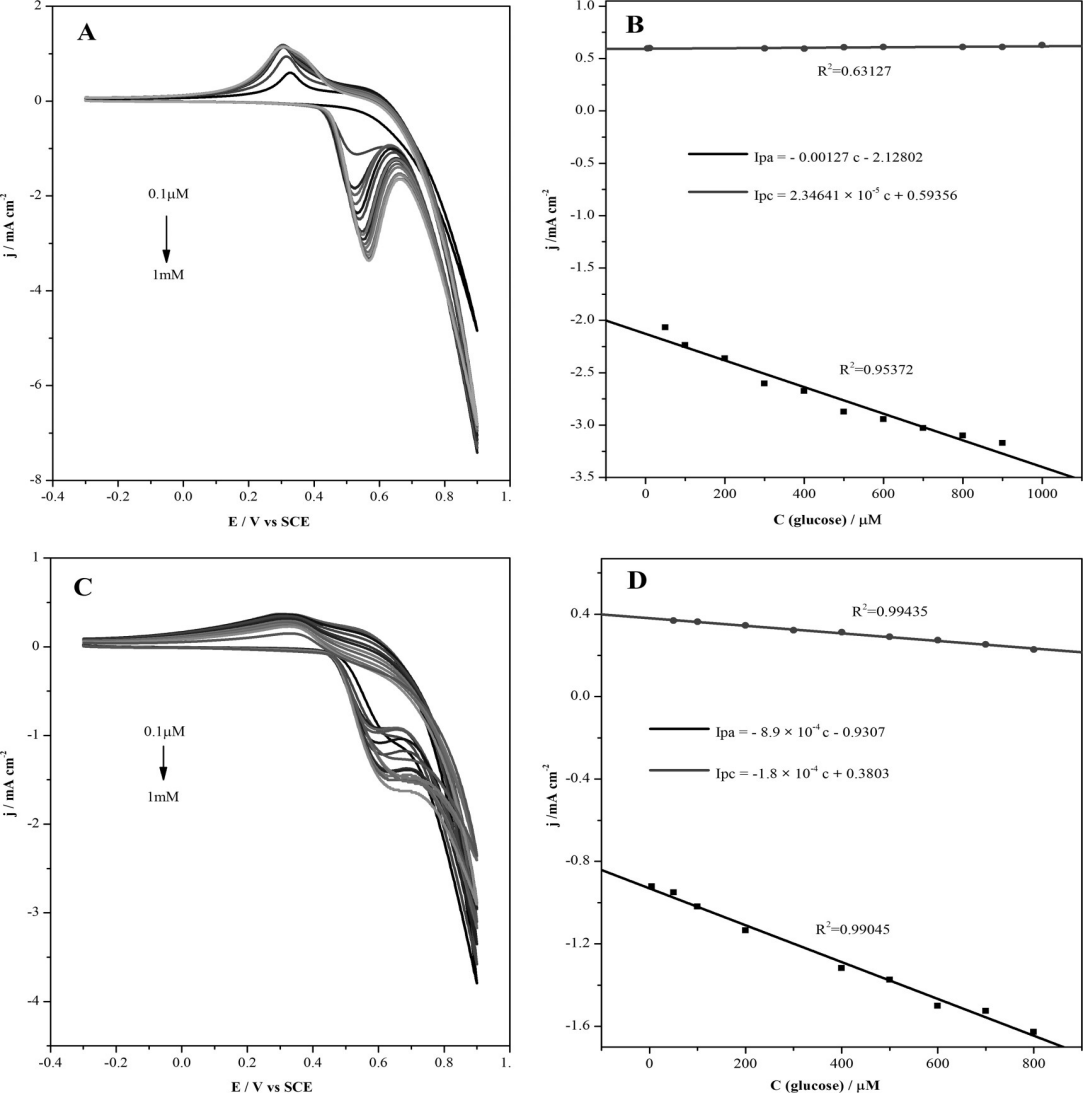
Electrocatalytic oxidation of Glucose on Cu@Ni CSNPs/Nafion/GCE and Cu@Ni CSNPs/N-GQDs/Nafion/GCE. CVs of modified Cu@Ni CSNPs/Nafion/GCE electrode at different glucose concentrations (**A**) and plot of peak current versus the different glucose concentrations (**B**); CVs of modified Cu@Ni CSNPs/N-GQDs/Nafion/GCE electrode at different glucose concentrations (**C**) and plot of peak current versus the different glucose concentrations (**D**).

CVs was used to study the relationship between current and glucose concentration. The CVs was obtained by placing modified electrodes in solutions containing different glucose concentrations at scan rate of 100 mV s^-1^. In Fig 4A, the CVs of modified Cu@Ni CSNPs/Nafion/GCE electrode has a glucose concentration range from 0.1 μM to 1 mM. Both anodic and cathodic currents are increased with the increasing of glucose concentration. Fig 4B shows the linear relationship between glucose concentration and current, *I*pa = -0.00127 c—2.12802 (R^2^ = 0.95372), *I*pc = 2.34641 ×10^−5^ c + 0.59356 (R^2^ = 0.63127). In Fig 4C, the CVs of the modified Cu@Ni CSNPs/N-GQDs/Nafion/GCE electrode has a glucose concentration range from 0.1 μM to 1 mM, both anodic and cathodic currents are increased with the increasing of glucose concentration. Fig 4D shows the linear relationship between glucose concentration and current, *I*pa = - 8.9 ×10^−4^ c—0.9307 (R^2^ = 0.99045), *I*pc = -1.8 ×10^−4^ c + 0.3803 (R^2^ = 0.99435). Compared with the above two sets of experiments, Cu@Ni CSNPs/N-GQDs/Nafion/GCE has better linearity and better determination of glucose concentration.

### The response of Cu@Ni CSNPs/Nafion/GCE and Cu@Ni CSNPs/N-GQDs/Nafion/GCE towards glucose

**Fig 5:**
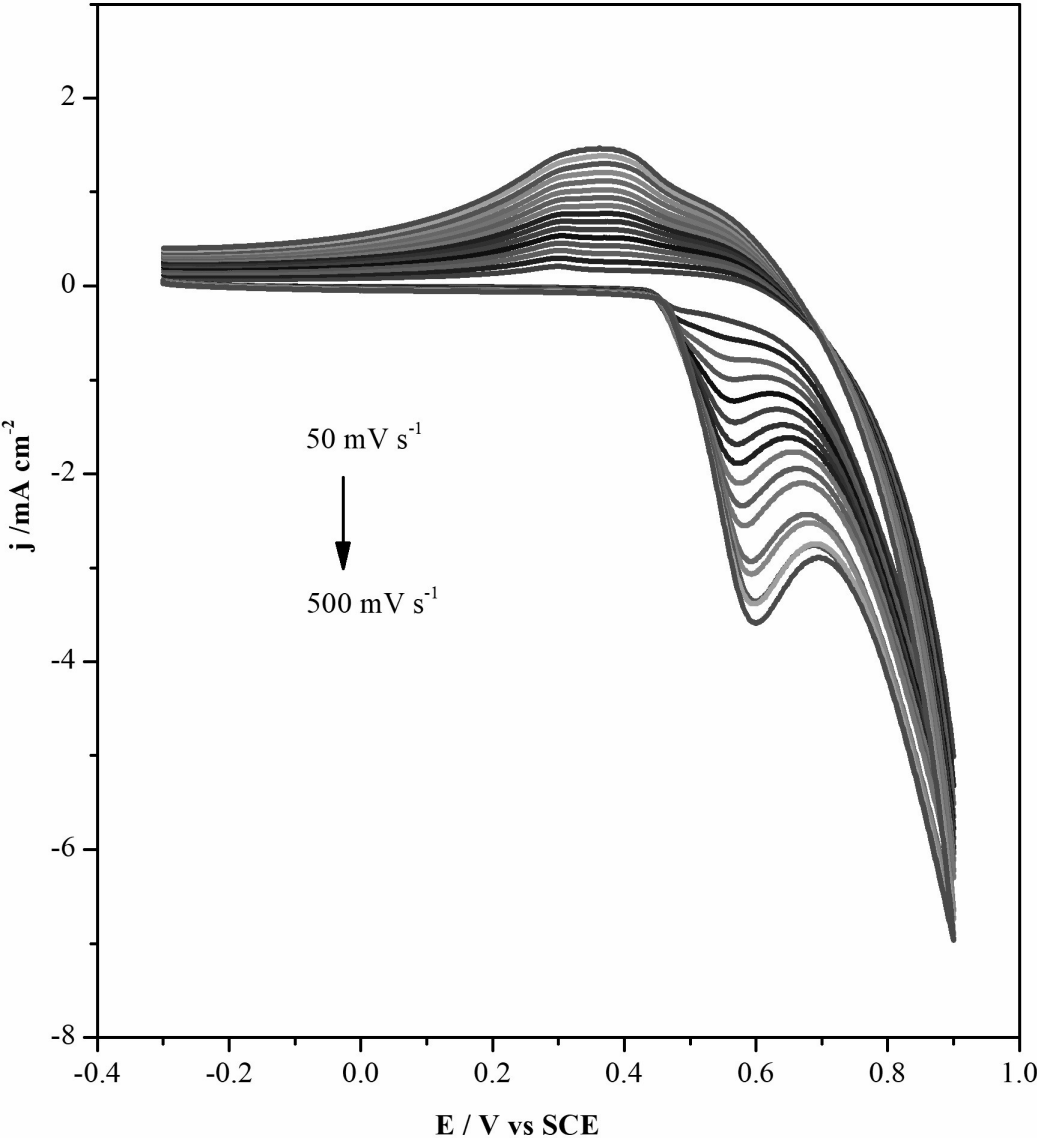
CVs of different scan rates of Cu@Ni CSNPs/N-GQDs/Nafion/GCE in 0.1 M NaOH containing 10 μM glucose.

The CVs of modified Cu@Ni CSNPs/N-GQDs/Nafion/GCE electrode at different scan rates in 0.1 M NaOH containing 10 μM glucose is shown in Fig 5. It can be seen from the figure that when the scan rate increasing, the current gradually increasing. The anodic potential range is from +0.5 ~ 0.7 V, which is the constant potential range for the next *i-t* curve test.

**Fig 6:**
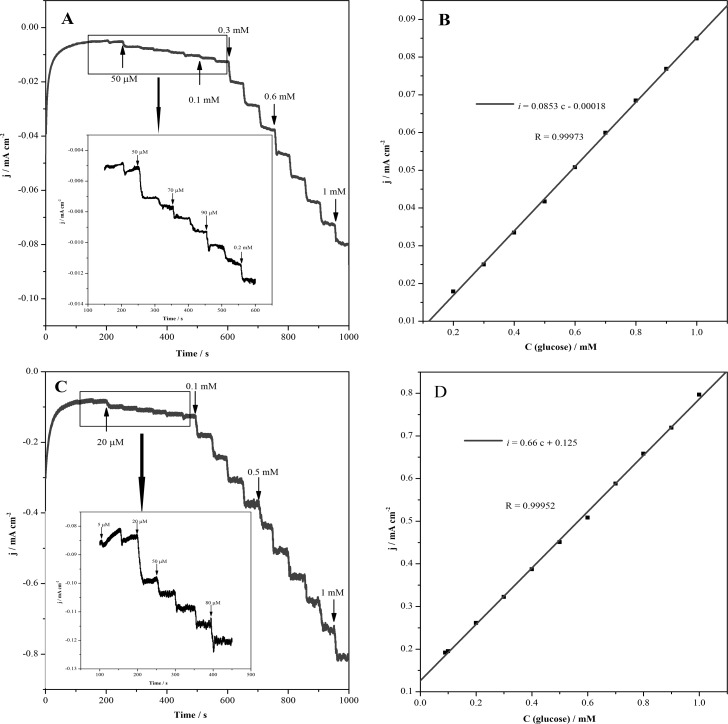
The response of Cu@Ni CSNPs/Nafion/GCE and Cu@Ni CSNPs/N-GQDs/Nafion/GCE towards glucose. Current-time responses at +0.6 V with an increasing glucose concentration every 50 s for the Cu@Ni CSNPs/Nafion/GCE (**A**) and the linear relationship between the catalytic current and glucose concentration (**B**); Cu@Ni CSNPs/N-GQDs/Nafion/GCE (**C**) and the linear relationship between the catalytic current and glucose concentration (**D**).

Under the optimal conditions, +0.6 V is selected as the constant potential in the range of anodic potential of +0.5 ~ 0.7 V. In Fig 6A–6D they show the current-time responses at +0.6 V with an increasing glucose concentration every 50 s for the Cu@Ni CSNPs/Nafion/GCE and Cu@Ni CSNPs/N-GQDs/Nafion/GCE, and the linear relationship between the catalytic current and glucose concentration. As shown in Fig 6A, it is the *i*-t curve of Cu@Ni CSNPs/Nafion/GCE at different glucose concentrations. The linear relationship between current and glucose concentration is shown in Fig 6B. The current response of the sensor exhibits a linear dependence on glucose concentration from 0.2 mM to 1 mM (*i* = 0.0853 c—0.00018, R = 0.99973). The detection limit of glucose using Cu@Ni CSNPs/Nafion/GCE is found to be 111.4 μM (S/N = 3) with the sensitivity of 85.3 μA mM^-1^ cm^-2^. As shown in Fig 6C, it is the *i*-t curve of Cu@Ni CSNPs/N-GQDs/Nafion/GCE at different glucose concentrations. The linear relationship between current and glucose concentration is shown in Fig 6D. The current response of the sensor exhibits a linear dependence on glucose concentration from 0.09 mM to 1 mM (*i* = 0.66 c + 0.125, R = 0.99952). The detection limit of glucose using Cu@Ni CSNPs/N-GQDs/Nafion/GCE is found to be 1.5 μM (S/N = 3) with the sensitivity of 660 μA mM^-1^ cm^-2^. From this, it can be clearly concluded that Cu@Ni CSNPs/N-GQDs/Nafion/GCE is more sensitive to glucose determination. This is because GQDs itself is conductive, and N-GQDs has more hole electron pairs due to nitrogen atoms have been successfully doped, which greatly improve the electrical conductivity. Glucose is catalyzed by Cu and Ni in the composites, and N-GQDs are coated on the surface of Cu@Ni CSNPs, which improves the electron mobility between the electrode and the electrolyte, so that the working electrode to detect glucose within a very short time, which greatly improves the sensitivity of the electrode and reduces the detection limit, providing conditions for real-time, rapid and accurate determination of glucose concentration.

### Selectivity of glucose by Cu@Ni CSNPs/N-GQDs/Nafion/GCE

The anti-interference property and selectivity for glucose determination are crucial in the development of glucose biosensors. Chemical species such as uric acid (UA), dopamine (DA), ascorbic acid (AA), and NaCl that easily oxidize are always present with glucose in human blood. In this study, interference experiments were detected by adding 0.1 mM interference component to a 0.1 M NaOH solution containing 0.5 mM glucose. As shown in S4A and S4B Fig, 0.5 mM glucose, 0.1 mM DA, 0.1 mM AA, 0.1 mM UA, 0.1 mM NaCl and 0.5 mM glucose were detected by Cu@Ni CSNPs/N-GQDs/Nafion/GCE at +0.6 V. Furthermore, other sugars may also affect the determination of glucose, such as sucrose, fructose, D-galactose, maltose, and so on. As shown in S5A and S5B Fig, 0.5 mM glucose, 0.1 mM maltose, 0.1 mM sucrose, 0.1 mM fructose, 0.1 mM D-galactose and 0.5 mM glucose were detected by Cu@Ni CSNPs/N-GQDs/Nafion/GCE at +0.6 V. The experimental results show that the current response of the interference components is very weak, indicating that low levels of sugar have very little affect for the glucose determination. Cu@Ni CSNPs/N-GQDs/Nafion/GCE has high selectivity for glucose determination.

## Summary

In summary, Cu@Ni CSNPs and Cu@Ni CSNPs/N-GQDs NCs were successfully synthesized by one-pot solvothermal method. Our experiments show that the electrochemical response of Cu@Ni CSNPs/N-GQDs/Nafion/GCE to glucose determination was the highest. Cu@Ni CSNPs/N-GQDs/Nafion/GCE has the advantages of low cost, high sensitivity and good selectivity. One more advantage of Cu@Ni CSNPs/N-GQDs/Nafion/GCE is its’ wide linear range is 0.09 ~ 1 mM. And the detection limit is 1.5 μM (S/N = 3), high sensitivity of 660 μA mM^-1^ cm^-2^, rapid response time (3 s). Comparing with the sensor based on Cu@Ni CSNPs modified working electrode, this novel nano-composite of Cu@Ni CSNPs/ N-GQDs NCs makes the sensor’s LOD more than 74 times lower, also has higher sensitivity and wider linear range. Furthermore, the interference components show insignificant interference in determination of glucose, and Cu@Ni CSNPs/N-GQDs/Nafion/GCE has high selectivity for glucose determination. The results indicate that the biosensor based on Cu@Ni CSNPs/N-GQDs/Nafion/GCE has potential application prospect in the determination of glucose, the application of GQDs in biosensor has a great prospect. It also indicates the great potential to apply this kind of nano composites in electrode modification and high sensitivity biomolecule detection.

## Supporting information

S1 Table XPS element content of N-GQDs and Cu@Ni CSNPs/N-GQDs NCs. (TIF)

S1 Fig XPS spectra of N-GQDs. (**A**) full spectrum, (**B**) C1s spectrum, (**C**) N1s spectrum, (**D**) O1s spectrum.
(TIF)

S2 Fig XPS spectra of Cu@Ni CSNPs/N-GQDs NCs. (**A**) C1s spectrum, (**B**) Cu2p spectrum, (**C**) Ni2p spectrum.
(TIF)

S3 Fig Mapping of Cu@Ni CSNPs/N-GQDs NCs (a), mapping-Cu (b), mapping-Ni (c).
(TIF)

S4 Fig Selectivity detection of glucose by Cu@Ni CSNPs/N-GQDs/Nafion/GCE with other chemical species. Amperometric response (**A**) and the histogram (**B**) of the Cu@Ni CSNPs/N-GQDs/GCE with successive addition of 0.5 mM glucose, 0.1 mM DA, 0.1 mM AA, 0.1 mM UA, 0.1 mM NaCl and 0.5 mM glucose in 0.1 M NaOH solution at +0.6 V, respectively.
(TIF)

S5 Fig Selectivity detection of glucose by Cu@Ni CSNPs/N-GQDs/Nafion/GCE with other sugars. Amperometric response (**A**) and the histogram (**B**) of the Cu@Ni CSNPs/N-GQDs/GCE with successive addition of 0.5 mM glucose, 0.1 mM maltose, 0.1 mM sucrose, 0.1 mM fructose, 0.1 mM D-galactose, 0.5 mM glucose in 0.1 M NaOH solution at +0.6 V, respectively.
(TIF)
