# Supplementary figures and images for: Novel bimetallic Cu/Ni core-shell NPs and nitrogen doped GQDs composites applied in glucose in vitro detection
Source: PLoS One. 2019 Jul 22;14(7):e0220005. doi: 10.1371/journal.pone.0220005 (PMC6645669; doi:10.1371/journal.pone.0220005)

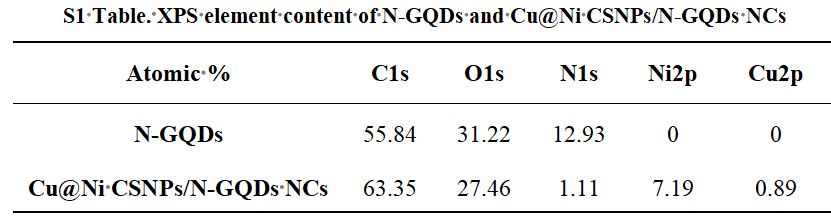

Supplement: S1 Table — (TIF) [file pone.0220005.s001.tif]

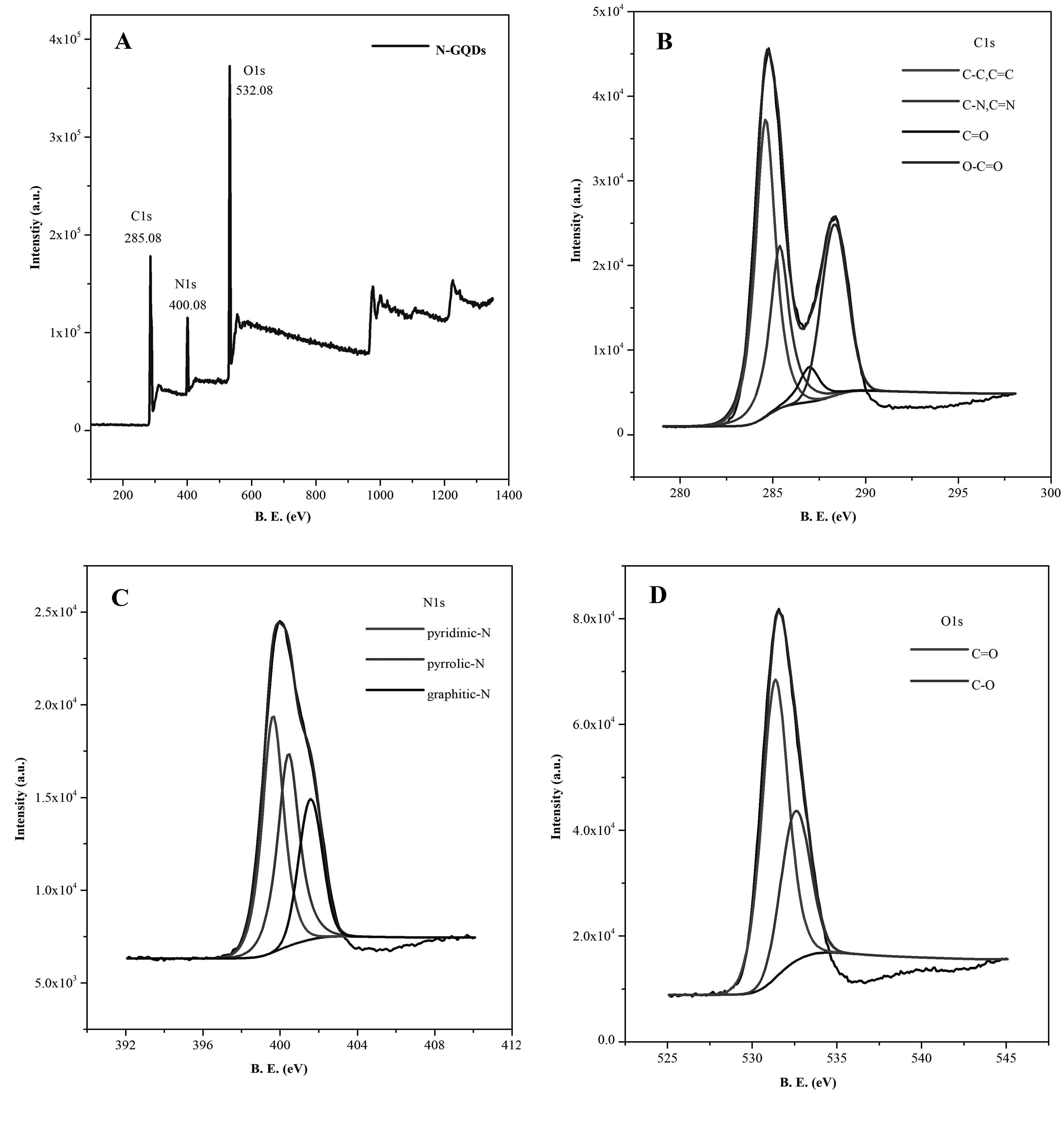

Supplement: S1 Fig — (A) full spectrum, (B) C1s spectrum, (C) N1s spectrum, (D) O1s spectrum. (TIF) [file pone.0220005.s002.tif]

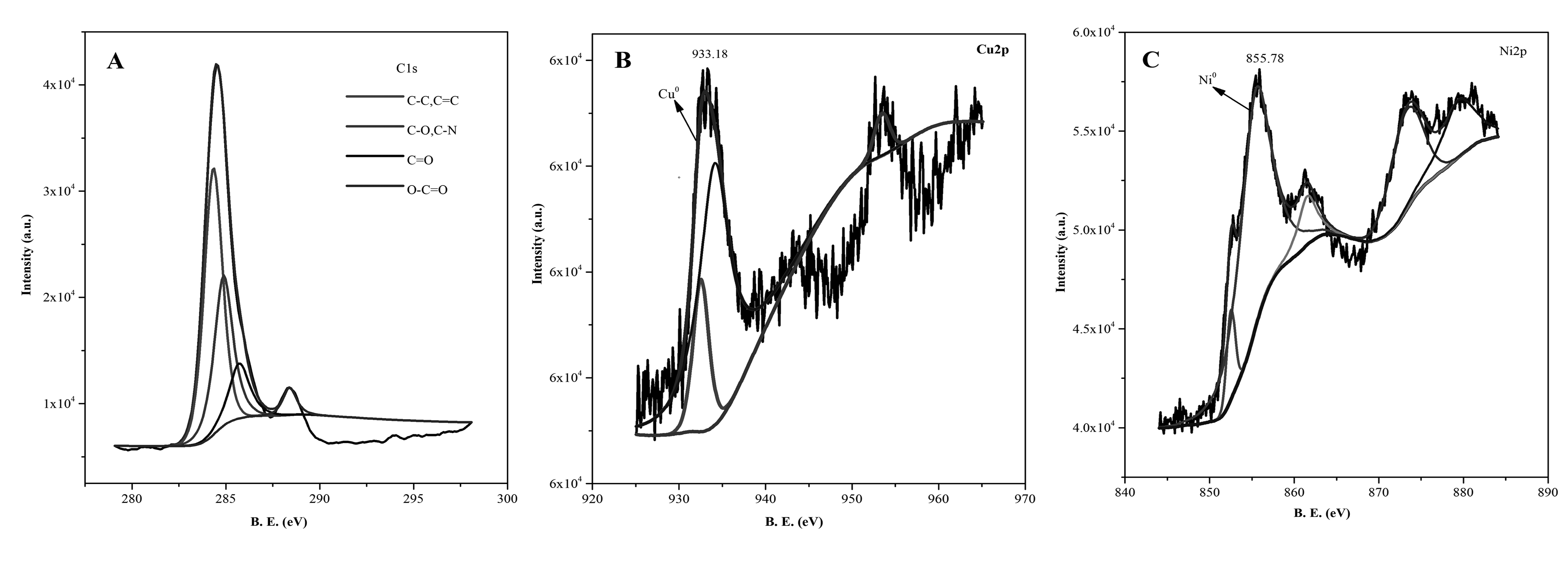

Supplement: S2 Fig — (A) C1s spectrum, (B) Cu2p spectrum, (C) Ni2p spectrum. (TIF) [file pone.0220005.s003.tif]

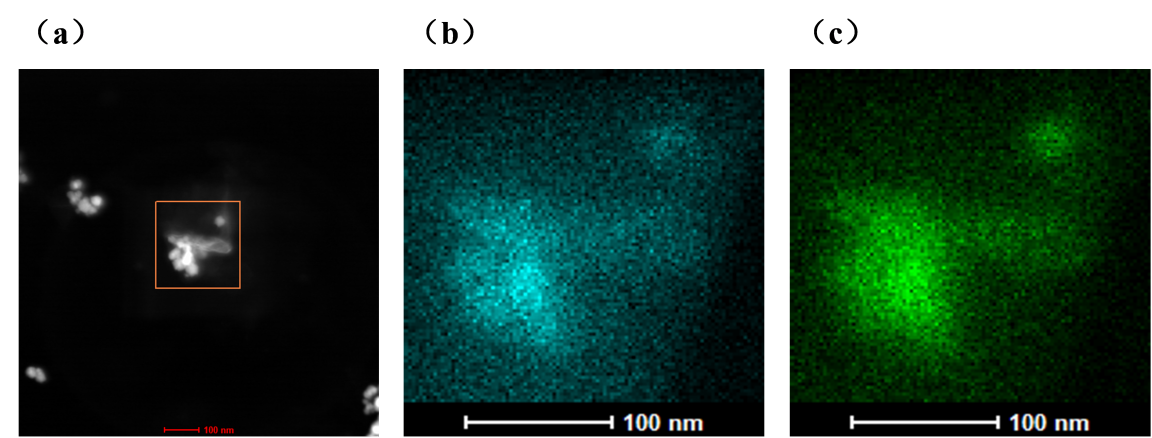

Supplement: S3 Fig — Mapping of Cu@Ni CSNPs/N-GQDs NCs (a), mapping-Cu (b), mapping-Ni (c). (TIF) [file pone.0220005.s004.tif]

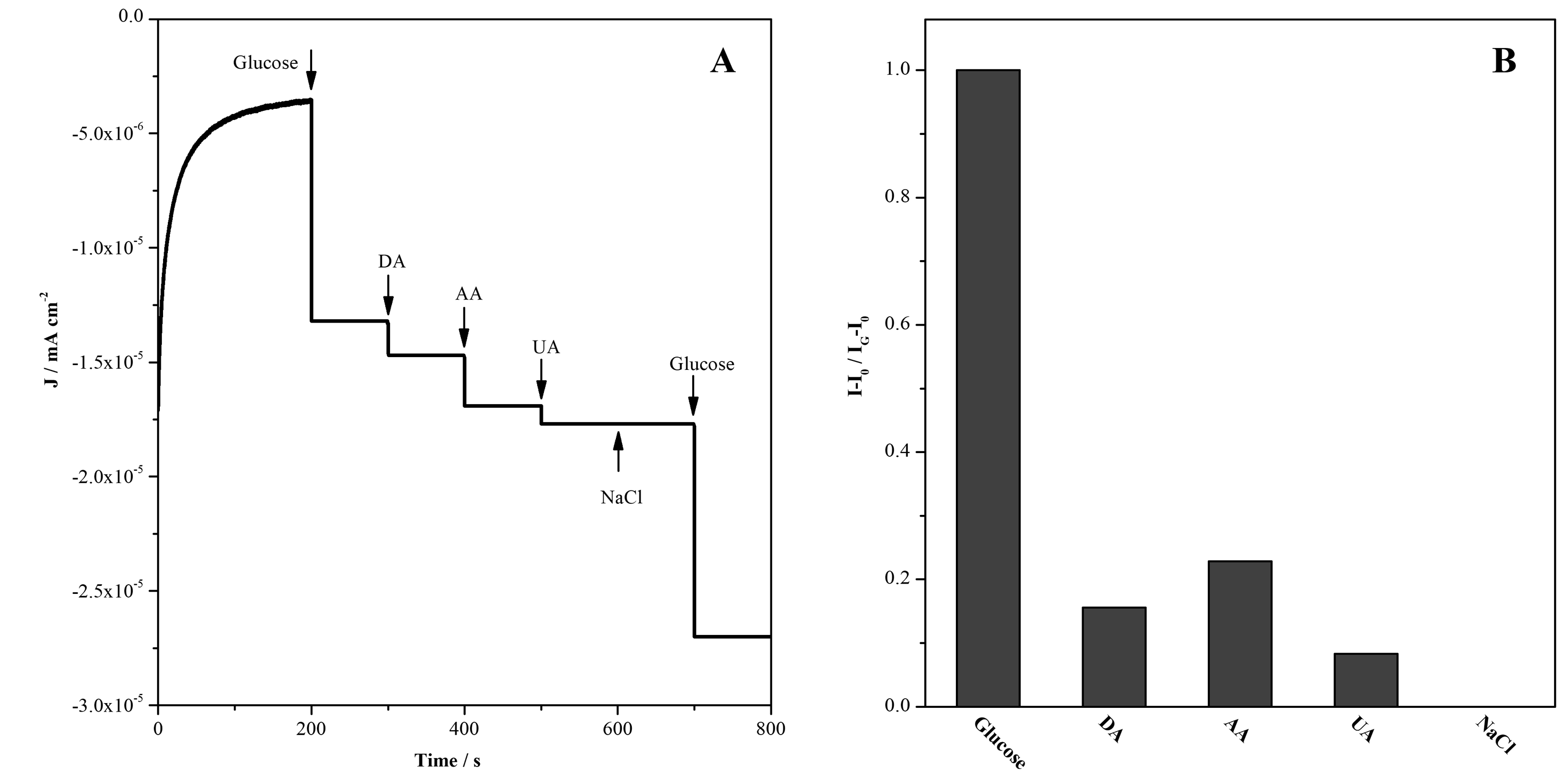

Supplement: S4 Fig — Amperometric response (A) and the histogram (B) of the Cu@Ni CSNPs/N-GQDs/GCE with successive addition of 0.5 mM glucose, 0.1 mM DA, 0.1 mM AA, 0.1 mM UA, 0.1 mM NaCl and 0.5 mM glucose in 0.1 M NaOH solution at +0.6 V, respectively. (TIF) [file pone.0220005.s005.tif]

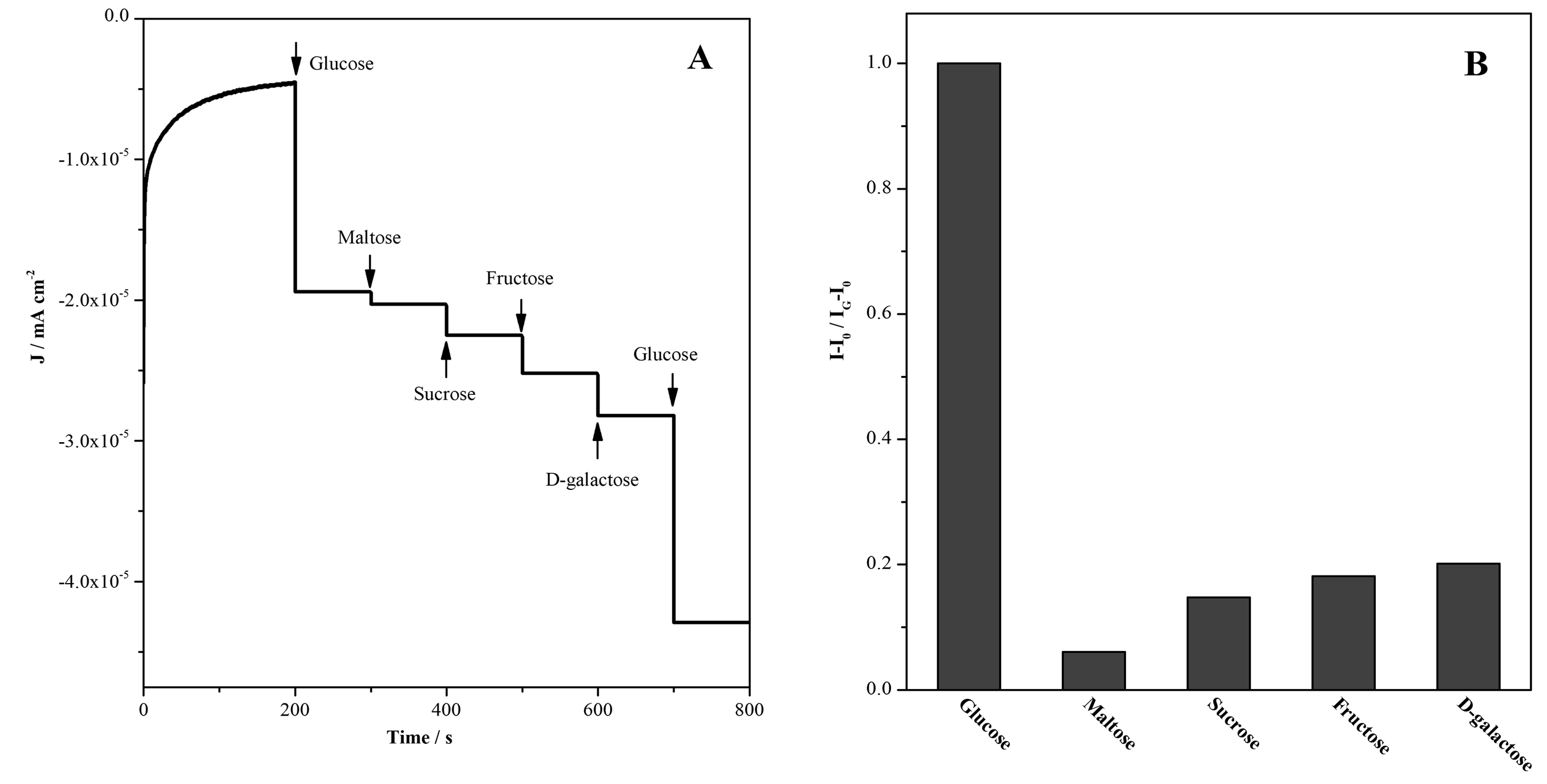

Supplement: S5 Fig — Amperometric response (A) and the histogram (B) of the Cu@Ni CSNPs/N-GQDs/GCE with successive addition of 0.5 mM glucose, 0.1 mM maltose, 0.1 mM sucrose, 0.1 mM fructose, 0.1 mM D-galactose, 0.5 mM glucose in 0.1 M NaOH solution at +0.6 V, respectively. (TIF) [file pone.0220005.s006.tif]
